# Naphthyridine derived colorimetric and fluorescent *turn off* sensors for Ni^2+^ in aqueous media

**DOI:** 10.1038/s41598-021-98400-2

**Published:** 2021-09-28

**Authors:** Abida Ashraf, Muhammad Islam, Muhammad Khalid, Anthony P. Davis, Muhammad Tayyeb Ahsan, Muhammad Yaqub, Asad Syed, Abdallah M. Elgorban, Ali H. Bahkali, Zahid Shafiq

**Affiliations:** 1 Institute of Chemical Sciences, Bahauddin Zakariya University, Multan, Pakistan; 2 Department of Chemistry, Khwaja Fareed University of Engineering and Information Technology, Rahim Yar Khan, 64200 Pakistan; 3 School of Chemistry, University of Bristol, Cantock’s Close, Bristol, BS8 1TS UK; 4 Department of Botany and Microbiology, College of Science, King Saud University, P.O. 2455, Riyadh, 11451 Saudi Arabia

**Keywords:** Environmental sciences, Natural hazards, Chemistry

## Abstract

Highly selective and sensitive 2,7-naphthyridine based colorimetric and fluorescence “Turn Off” chemosensors (L1-L4) for detection of Ni^2+^ in aqueous media are reported. The receptors (L1-L4) showed a distinct color change from yellow to red by addition of Ni^2+^ with spectral changes in bands at 535–550 nm. The changes are reversible and pH independent. The detection limits for Ni^2+^ by (L1-L4) are in the range of 0.2–0.5 µM by UV–Visible data and 0.040–0.47 µM by fluorescence data, which is lower than the permissible value of Ni^2+^ (1.2 µM) in drinking water defined by EPA. The binding stoichiometries of L1-L4 for Ni^2+^ were found to be 2:1 through Job’s plot and ESI–MS analysis. Moreover the receptors can be used to quantify Ni^2+^ in real water samples. Formation of test strips by the dip-stick method increases the practical applicability of the Ni^2+^ test for “in-the-field” measurements. DFT calculations and AIM analyses supported the experimentally determined 2:1 stoichiometries of complexation. TD-DFT calculations were performed which showed slightly decreased FMO energy gaps due to ligand–metal charge transfer (LMCT).

## Introduction

The development and synthesis of chemosensors for selective and sensitive detection of heavy and transition metal ions is an active area of present day research due to their substantial effects on the environment and biological systems^1–3^. The sensing of metal ions in aqueous media is a complex task due to the presence of competitive interactions between the solvent and guest for receptor binding sites^4,5^. Among the various transition metals, nickel is an important element due to its widespread use in industry (Ni–Cd batteries), in ceramics and magnetic components for computers, metallurgical processes (electroplating), rods for arc welding, surgical and dental prostheses, and pigments for paints^6–8^. Nickel has a significant role in various enzymatic activities such as acireductone dioxygenases, carbon monoxide dehydrogenases, and catalysts for hydrogenation. It is also used as an essential trace element in biological systems, with significance in the biosynthesis and metabolism of some plants and microorganisms. However it is a toxic metal from a bio-medical point of view, as it can be easily absorbed by organs such as spleen liver, kidney etc. and may cause lung cancer, nasopharyngeal carcinoma, asthma, pneumonitis, and disorders of the central nervous system in humans. Deficiency or excessive levels of nickel also affect the life of many prokaryotic and eukaryotic organisms^9–13^.

Considering all these facts, the selective monitoring of nickel is very important in environmental, biological and industrial samples. Various analytical methods such as flame atomic absorption spectrometry-electro thermal atomization (FAS-ETA), atomic absorption spectrometry (AAS), and inductively coupled plasma atomic emission spectrometry (ICP-AES) are widely used for detection of metal ions^14–19^. However, most of the methods need trained operators, sophisticated equipment and tedious sample preparation procedures, so there is still a need for simple, efficient and cost-effective methods for the micro level detection of heavy metals ions.

In recent years colorimetric, ratiometric, potentiometric and fluorescence sensors have gained attention for the selective detection of metal ions such as Ni^2+^ in biological and environmental samples. Colorimetric chemosensors show a distinct visible color change on selectively binding with a specific analyte without the use of expensive equipment, while the fluorescent sensors show fast responses towards analytes through fluorescence quenching or enhancement. These sensors have some drawbacks like poor solubility of probe, lack of selectivity, low sensitivity, and serious interference by other metals^20^.

**Figure 1:**
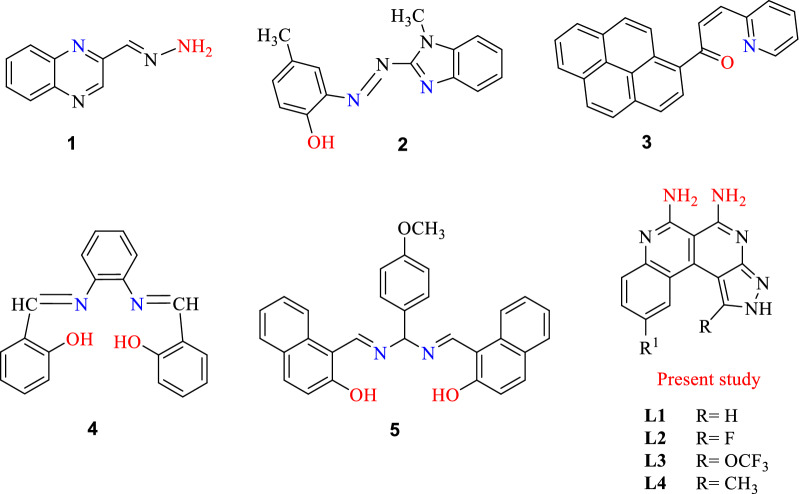
Some structural motifs employed in Nickel sensors, and L1-L4 from the present study.

Colorimetric sensors seem to be especially promising due to low cost, rapid detection and ease of use when compared to classical techniques as well as fluorescent sensors. Several organic molecules have been reported which showed colorimetric and fluorescent sensing of Ni^2+^ but few were capable of parts per million level detection. The signals from absorption and emission changes of light by chromophores or fluorophores provide information about the mechanism of sensing of metal cation (Ni^2+^) by electron transfer (ET), charge transfer (CT), photoinduced electron transfer (PET), excited state intramolecular proton transfer (ESIPT) mechanism^21,22^. In recent times, the Goswami group reported a quinoxaline **1** based ratiometric chemosensor for the naked eye detection of Ni^2+^ in CH_3_CN media^23^. Sarkar et al. developed benzimidazole based **2** as a ratiometric and colorimetric chemosensor for Ni^2+^ in DMSO^24^. Prabhu and co-workers developed a selective fluorescent turn on chemosensor for Ni^2+^ based on pyrene-conjugated pyridine^13^ **3**. Biswajit Chowdhury et al. developed a Salen type Schiff base **4** colorimetric chemosensor with fluorescent enhancement for Ni^2+^ ^25^. S. Santhi et al. developed the phenylmethanediamine based **5** as a colorimetric and turn off fluorescent sensor for detection of Co^2+^, Ni^2+^ and Cu^2+^ in aqueous methanol solution^26^ (Fig. 1).

Benzo[*c*]pyrazolo[2,7]naphthyridines and its derivatives have gained much attention in last few decades because they are biologically active alkaloids isolated from marine organisms such as perlolidine^27^ (cellulose digestive inhibitor), subarine^28^ (anti-HIV activity), meridine^29^ (cytotoxic activity), amphimedine^30,31^ (top isomerase II) inhibitor) and PDK-1 inhibitors^32^.

In continuation of our research work in the field of molecular recognition, we herein report benzo[*c*]pyrazolo[2,7]naphthyridine-5,6-diamine based efficient colorimetric and/or *fluorescence off* sensors that can detect Ni^2+^ with sensitivity and selectivity in aqueous solutions. Many researchers have utilized amino group containing sensors like diaminonaphthalene^33^, 1,8-naphthyridine-amine^34^, 1,8-naphthyride-2-acetoamide^35^, diaminophenazine and 1,2-diaminoanthracene-dione^36^ to sense different metal ions like Cu^2+^, Hg^2+^, Fe^3+^, Al^3+^. Here we report sensors in which vicinal amino groups are placed on the benzo[*c*]pyrazolo[2,7]naphthyridine skeleton. Diamines L1-L4 serve as sensors for Ni^2+^ in aqueous solutions, utilizing the two amino groups as binding sites for the metal ion. The receptors detect the cation Ni^2+^ by both Chelation Enhanced Fluorescence Quenching (CHEFQ and an instant change in color from yellow to red. The chemosensors L1-L4 could be used as practical sensors for quantitative determination of nickel at ppm level in real water samples and also used in test kits to observe the color changes for Ni^2+^ by a dip stick method. Importantly the sensing potential of these derivatives can be tuned by the nature of the substituents R, i.e. electron donating (CH_3_) and electron attracting (F, OCF_3_), which affect the sensing properties. Quantum chemical computations (DFT) were performed to get detailed insight on the interaction between L4 and Ni^2+^ and are in good agreement with experimental findings.

## Results and discussion

The benzo[c]pyrazolo[2,7]naphthyridine based chemosensors were synthesized as shown in Scheme S1. The isatins **6** react with malononitrile **7** via Knoevenogel condensation to form arylidenes. The formed arylidenes are then reacted with 3-amino-5-methylpyrazole **8** to synthesize spiro-intermediates which then undergo basic hydrolysis, cyclization, decarboxylation and aromatization to form target naphthyridine receptors (L1–L4)^37^.

### Spectrophotometric studies of L1–L4

#### UV–visible study

**Figure 2:**
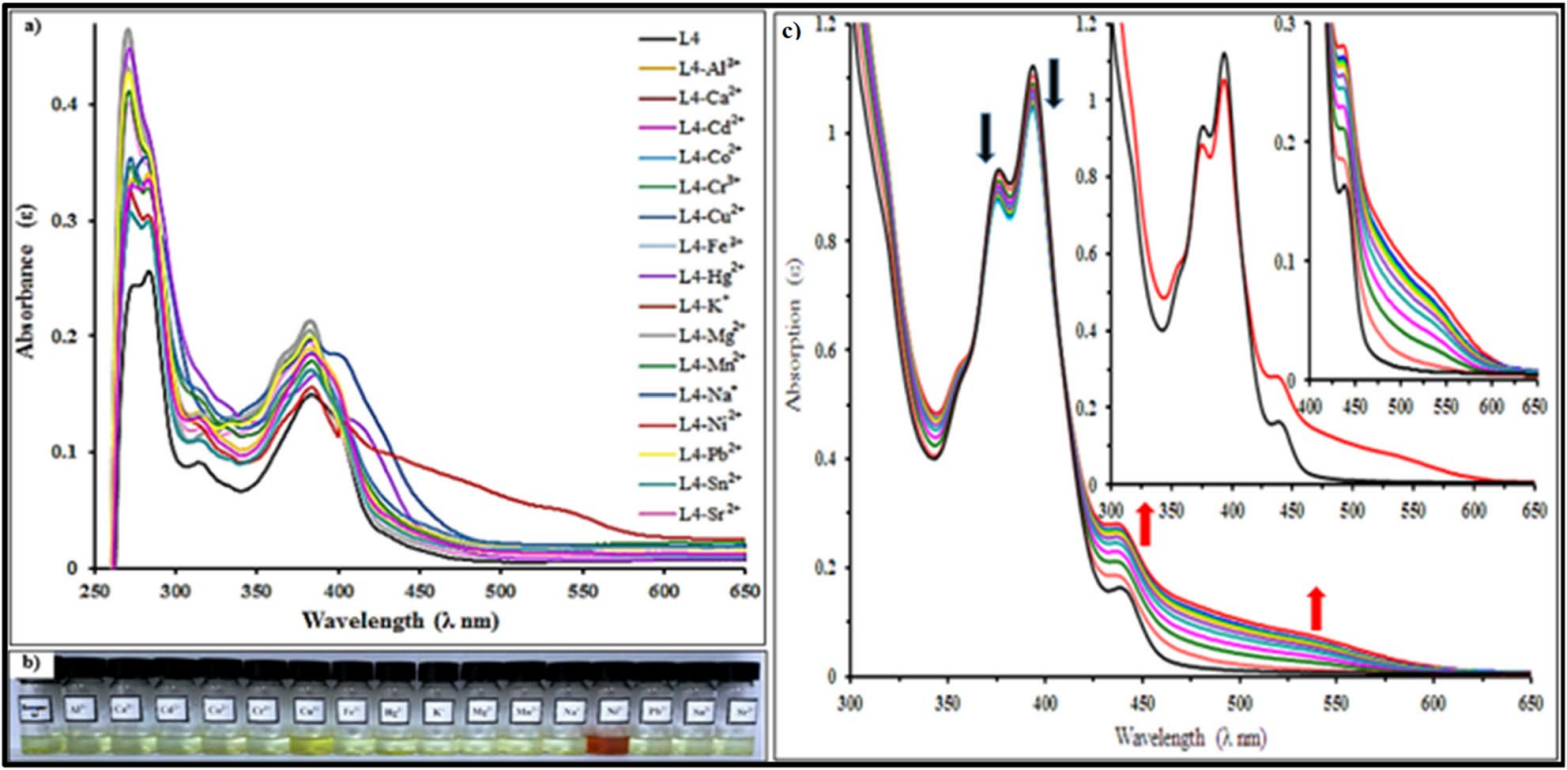
(**a**) Absorption spectral changes of L4 (20 µM) in the presence of different metal ions in DMSO–H_2_O (v/v 1:2, HEPES buffer pH = 7.4), (**b**) Visual colorimetric response of receptor L4 upon addition of one equivalent various metal ions, (**c**) Absorbance titration spectra of receptor L4 (20 µM) in the presence of various concentrations of Ni^2+^ (0–12 µM) in DMSO*–*H_2_O (v/v 1:2, HEPES buffer, pH = 7.4).

Ligands L1–L4 were investigated as chemosensors for various metal ions (Al^3+^, Ca^2+^, Cd^2+^, Co^2+^, Cr^3+^, Cu^2+^, Fe^3+^, Hg^2+^, K^+^, Mg^2+^, Mn^2+^, Na^+^, Ni^2+^, Pb^2+^, Sr^2+^ and Sn^2+^) by UV–visible spectroscopy. The preliminary colorimetric experiments involved addition of one equivalent of metal ions (1 × 10^−3^ M) to solutions of L1–L4 (1 × 10^−3^ M) in DMSO–H_2_O (v/v 1:2), HEPES buffer of pH = 7.4 at room temperature. The addition of Ni^2+^ resulted in distinct visual color changes from yellow to red, but no color change was observed for other metal ions (Al^3+^, Ca^2+^, Cd^2+^, Co^2+^, Cr^3+^, Cu^2+^, Fe^3+^, Hg^2+^, K^+^, Mg^2+^, Mn^2+^, Na^+^, Pb^2+^, Sr^2+^ and Sn^2+^) Fig. 2a-b.

The binding interaction of L1–L4 with different metal ions was further monitored by investigating UV–visible absorption spectral changes as indicated in Table 1.

**Table 1:** UV–visible spectral bands of receptor L1-L4 with addition of nickel (II).

Receptor	UV–visible spectral band (nm)	Nickel addition (nm)
L1	273, 283, 398, 440	535
L2	274, 286, 384, 443	538
L3	261, 287, 380, 450	550
L4	273, 285, 396, 438	537

The UV–visible spectra of model receptor L4 showed a remarkable bathochromic shift in absorption spectrum at 537 nm which is in good agreement with the color change. This may possibly be ascribed to fast metal–ligand binding kinetics and the high thermodynamic affinity of Ni^2+^ for N-donor ligands^38^. The other examined metal ions did not cause any distinct spectral changes in the UV–visible spectrum at 537 nm under identical conditions. A similar pattern of absorption spectral changes were observed for L1-L3 (Figs. S1–S3).

The coordination between receptor L4 and Ni^2+^ was further demonstrated by UV–visible absorption spectral titrations involving sequential addition of Ni^2+^ aliquots (0–12 µM) to L4 (20 µM). It was observed that the intensity of the absorption bands at 537 nm and 438 nm increased while the absorption bands at 396 nm and 376 nm began to decrease until reaching limiting values. Moreover the emergence of isosbestic points at 365 nm and 410 nm during spectral titrations indicated the formation of stable complexes with definite stoichiometric ratios between L4 and Ni^2+^ ^23^ (Fig. 2c). Similar coordination behavior was observed for L1-L3 (Figs. S4–S6). These results suggest that receptors L1-L4 could be employed as colorimetric and ratiometric sensors for Ni^2+^ and discriminating against different transition metal ions (Fe^3+^, Cu^2+^, Co^2+^, Pb^2+^, Hg^2+^) which are normally difficult to differentiate.

#### Fluorescence study

**Figure 3:**
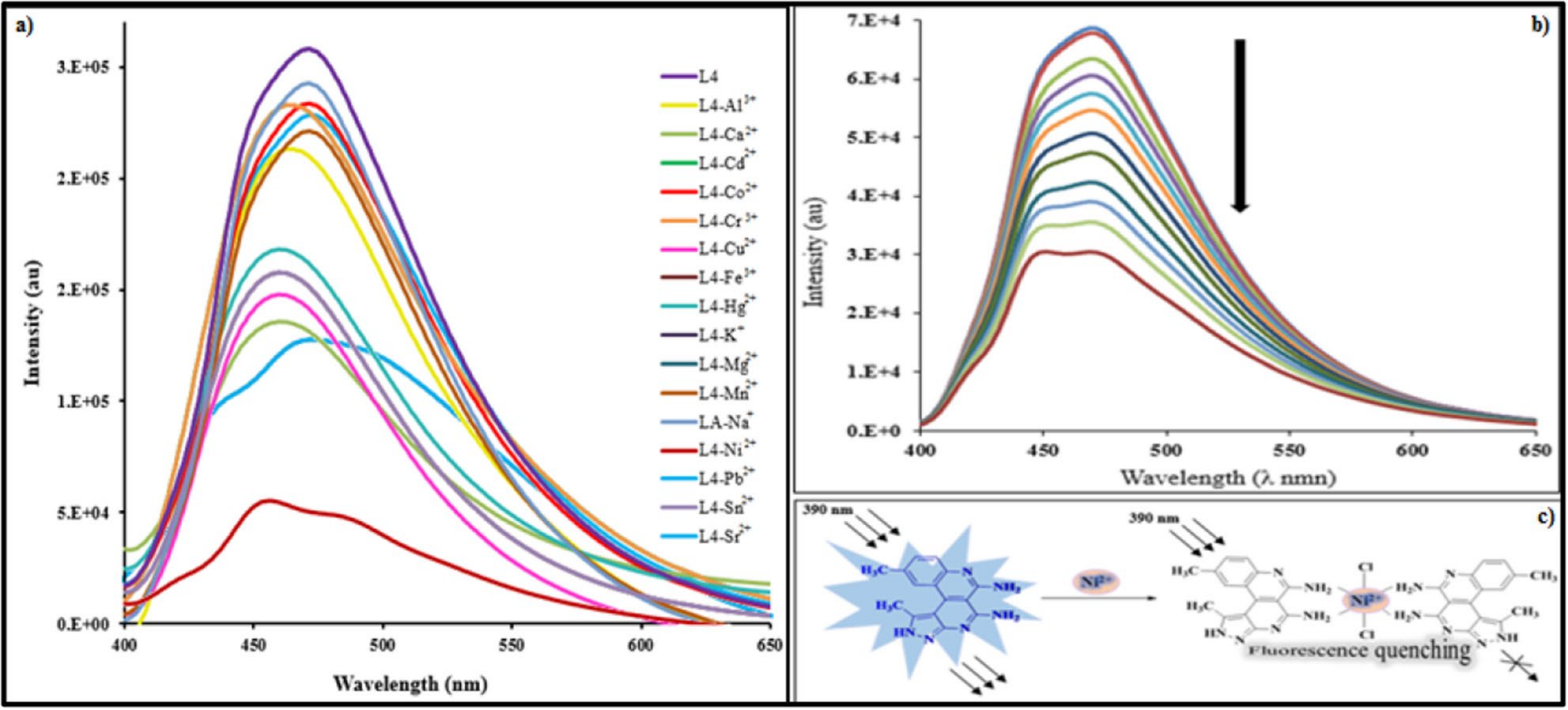
(**a**) Fluorescence spectral changes of L4 (20 µM) in the presence of different metal ions in DMSO–H_2_O (v/v 1:2, HEPES buffer pH = 7.4), (λ_ex_ = 390 nm), (**b**) Fluorescence spectra of receptor L4 (20 µM) in the presence of various concentrations of Ni^2+^ (0–12 µM) HEPES buffer, pH = 7.4 (λex = 390 nm), (**c**) Proposed detection mechanism of receptor L4 to Ni^2+^.

Photophysical complexation studies of L1-L4 at room temperature with metal ions were also performed with fluorescence spectroscopy. The fluorescence spectra of L4 showed an emission band at 470 nm (λ_ex_ = 390 nm) in DMSO–H_2_O (v/v 1:2, HEPES buffer, pH = 7.4). Amongst addition of different metal ions (Al^3+^, Ca^2+^, Cd^2+^, Co^2+^, Cr^3+^, Cu^2+^, Fe^3+^, Hg^2+^, K^+^, Mg^2+^, Mn^2+^, Na^+^, Ni^2+^, Pb^2+^, Sr^2+^ and Sn^2+^), the emission band at 470 nm underwent significant quenching with Ni^2+^ in comparison with the other metal ions (Fig. 3a). Similar patterns of fluorescence spectral changes were observed for L1-L3 (Figs. S7–S9).

The coordination behavior of L4 was examined via fluorescence titrations at room temperature. The sequential addition of Ni^2+^ (0–12 µM) to receptor L4 caused a substantial decrease of emission intensity at 470 nm, indicating “*turn-off*” behavior of the receptor (Fig. 3b). The rigid and planar structure of L4 molecule makes it a highly fluorescent compound. However when chelation occurs between the NH_2_ groups of receptor L4 and Ni^2+^, the amino groups lose their ability to donate electron density into the fluorophore due to ligand–metal charge transfer (LMCT) which causes the Chelation Enhancement Quenching Effect (CHEQ) and quenching of emission potential^39^ (Fig. 3c). Similar coordination behavior was observed for L1-L3 (Figs. S10–S12).

#### Binding stoichiometry, association constant and detection limit

The binding stoichiometry of the complexes were further explored by Job’s continuous variation method^40^ by plotting mole fraction versus changes in absorption intensity at 535 nm for L1, 538 nm for L2, 550 nm for L3 and 537 nm for L4, respectively. The Job’s plots (Fig. S13) indicate maximum values at 0.7 corresponding to the formation of complexes with 2:1 stoichiometry between L1–L4 and Ni^2+^.

The association constants *K*_*a*_ of receptors L1–L4 with Ni^2+^ were determined by analysis of the UV–visible and fluorescence data using the Benesi–Hildebrand equation^41^

$$\frac{1}{{\left( {A - A_{{\text{o}}} } \right)}} = \frac{1}{{\left\{ {\left( {A_{\max } - A_{{\text{o}}} } \right)K_{a} [{\text{Ni}}^{2 + } ]^{n} } \right\}}} + \frac{1}{{\left( {A_{\max } - A_{{\text{o}}} } \right)}}$$

(Figure S14, S15) and are listed in Table S1.

The association constant values are in range of those 10^3^–10^6^ reported for Ni^2+^ sensing chemosensors^42^. The trend of *K*_*a*_ values shows that L4-Ni^2+^ complex is stronger than the other receptor complexes. Moreover the association constants obtained by UV–visible data were on the same order of trend for as those obtained from fluorescence data i.e. L4-Ni^2+^  > L3-Ni^2+^  > L2-Ni^2+^  > L1-Ni^2+^.

The detection limits of L1–L4 for Ni^2+^ as colorimetric sensors were determined by naked eye, absorption and fluorescence spectral changes Table 2.

**Table 2:** Detection limit of receptor L1-L4 with nickel.

Receptor	Detection limit (3S_B_/S) M		Detection limit (Naked eye) M
	Absorbance	Emission	
L1	5.62 × 10^−7^	4.76 × 10^−7^	1 × 10^−5^
2	5.56 × 10^−7^	1.19 × 10^−7^	1 × 10^−5^
L3	3.88 × 10^−7^	6.61 × 10^−8^	1 × 10^−5^
L4	2.43 × 10^−7^	4.03 × 10^−8^	1 × 10^−6^

**Figure 4:**
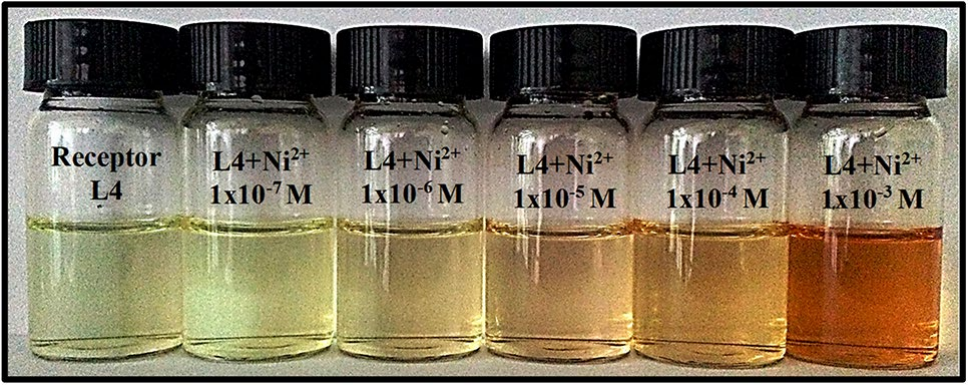
Naked eye detection limit for receptor L4.

For naked eye detection, the receptor L4 showed a distinct color change at a minimum concentration of 1 × 10^−6^ M for Ni^2+^ (Fig. 4/S16). Moreover, the detection limits determined by absorption and fluorescence spectral changes on the basis of 3S_B_/S ^43^ for L4 and Ni^2+^ were found to be 2.43 × 10^−7^ M and 4.03 × 10^−8^ M respectively. These values are lower than EPA drinking water guidelines (1.2 × 10^−6^ M for Ni^2+^ ^44^) and revealed that L4 is highly efficient in sensing Ni^2+^ even at minute levels.

### Proposed sensing mechanism

#### ESI–MS, IR and ^1^HNMR titrations

The coordination mechanism of L4 to Ni^2+^ was explored by ESI–MS and IR titration experiments. The ESI mass spectra of L4 showed a peak at m/z: 279.17 [M + H]^+^ corresponding to [L4 + H]^+^. After titration of L4 with Ni^2+^ a signal at *m/z*: 685.58 for [2L4 + Ni + Cl_2_ + H]^+^ (calculated *m/z* = 684) indicated the formation of a 2:1 stoichiometry complex between L4 and Ni^2+^ (Fig. S17).

**Figure 5:**
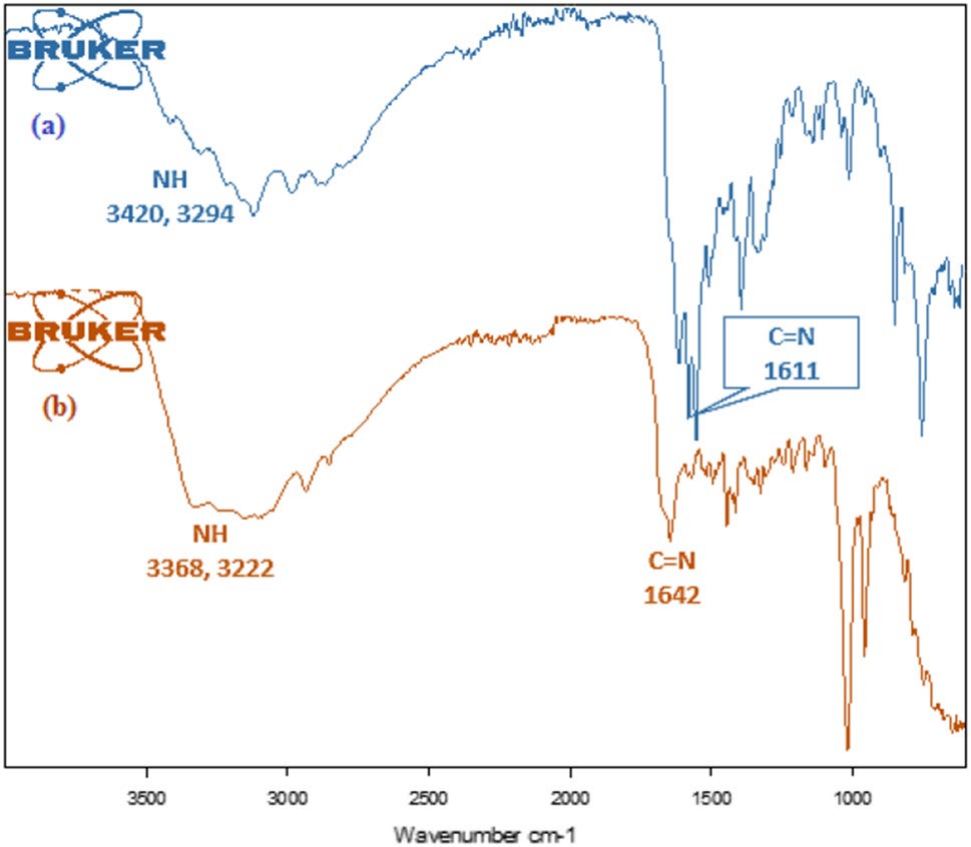
IR spectra of (**a**) receptor L4 and (**b**) receptor L4–Ni^2+^ complex.

**Scheme 1:**
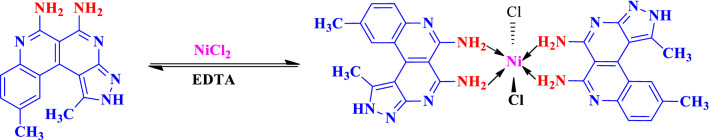
The plausible sensing mechanism of Ni^2+^ and with L4.

FT-IR titrations were performed by using a Bruker Alpha FT-IR. Figure 5 shows a comparison of the IR spectra of L4 before and after the addition of Ni^2+^. The sharp peaks present at 3420, 3294 and 3109 cm^−1^ due to NH stretching frequencies in free receptor L4 were broadened by adding Ni^2+^, suggesting the involvement of NH_2_ group in coordination with Ni^2+^ to form complex^45,46^. To further investigate the interaction behavior between L4 and Ni^2+^, we carried out ^1^HNMR titrations but these were not successful due to paramagnetic property of L4-Ni^2+^ complex^42^. The plausible sensing mechanism of L4 for Ni^2+^ is shown in Scheme 1.

#### Metal ion selectivity

**Figure 6:**
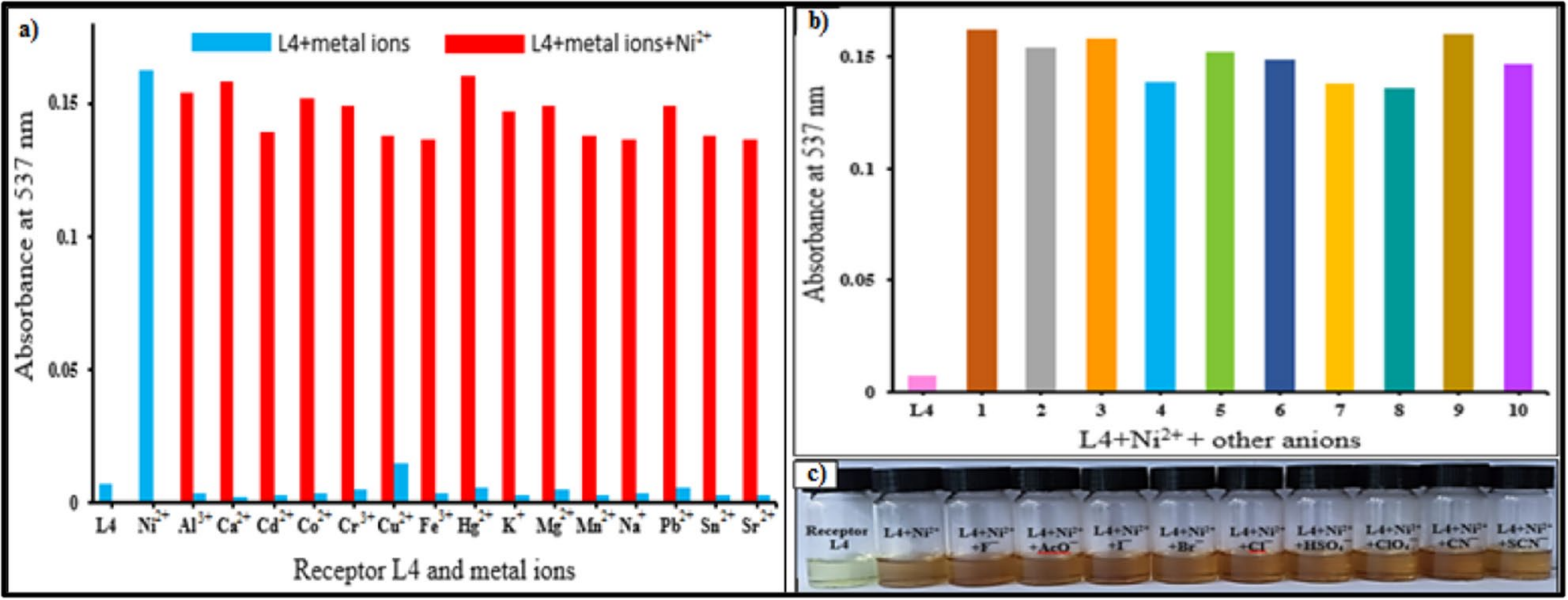
(**a**) Absorbance responses of L4 (20 µM) in the presence of Ni^2+^ (10 µM) with 10 equivalents of various metal ions in DMSO*–*H_2_O (v/v = 1:2) HEPES buffer solutions at pH = 7.4, (**b**) Absorbance at 537 nm of receptor L4 (20 µM) in the presence of Ni^2+^ (10 µM) in the presence of 10 equivalents of various anions in DMSO–H_2_O (v/v = 1:2) HEPES buffer solutions at pH = 7.4, (**c**) The color changes of L4 upon addition of Ni^2+^ and various anions (1–10).

An important feature of receptor L4 is its selectivity towards analytes. This was examined by competitive titration experiments (Fig. 6a). The intensity of the absorption band at 537 nm due to complex formation of L4-Ni^2+^ is not disturbed at all in the presence of other metal ions (Al^3+^, Ca^2+^, Cd^2+^, Co^2+^, Cr^3+^, Cu^2+^, Fe^3+^, Hg^2+^, K^+^, Mg^2+^, Mn^2+^, Na^+^, Pb^2+^, Sr^2+^ and Sn^2+^). Thus the receptor L4 shows excellent binding affinity for Ni^2+^, which should hold in physiological samples where Cu^2+^, Co^2+^, Fe^3+^, Hg^2+^ and Pb^2+^ usually coexist with analyte. This distinct selectivity for Ni^2+^ may be due to matching of the geometry of the receptor with the ionic radius of Ni^2+^ ^13^.

The UV–visible absorption spectra of the L4-Ni^2+^ complex with various anions was recorded to check the stability of complex. No change in the absorption band at 537 nm was observed (Fig. 6b-c). This clearly indicates that the stability of the complex is unaffected by the presence of various anions.

#### pH effect study

In order to investigate the effect of pH on the absorption response of receptor L4 to Ni^2+^, a series of solutions with pH values ranging from (2.0 – 12.0) were prepared (Fig. S18).

At pH 2.0–3.0, the receptor L4 shows no substantial response to Ni^2+^ in absorption spectroscopy. The absorption of the L4-Ni complex at 537 nm is maximum and constant in pH range 7.0 – 8.0. Above pH 8.0, absorbance decreased gradually. The results suggest that biological and environmental applications at physiological pH should be feasible. The color of L4– Ni^2+^ complex remained red between pH 4–11, which indicated that Ni^2+^ could be clearly detected over a wide range of pH 4–11.

#### Reversibility of receptor L4

**Figure 7:**
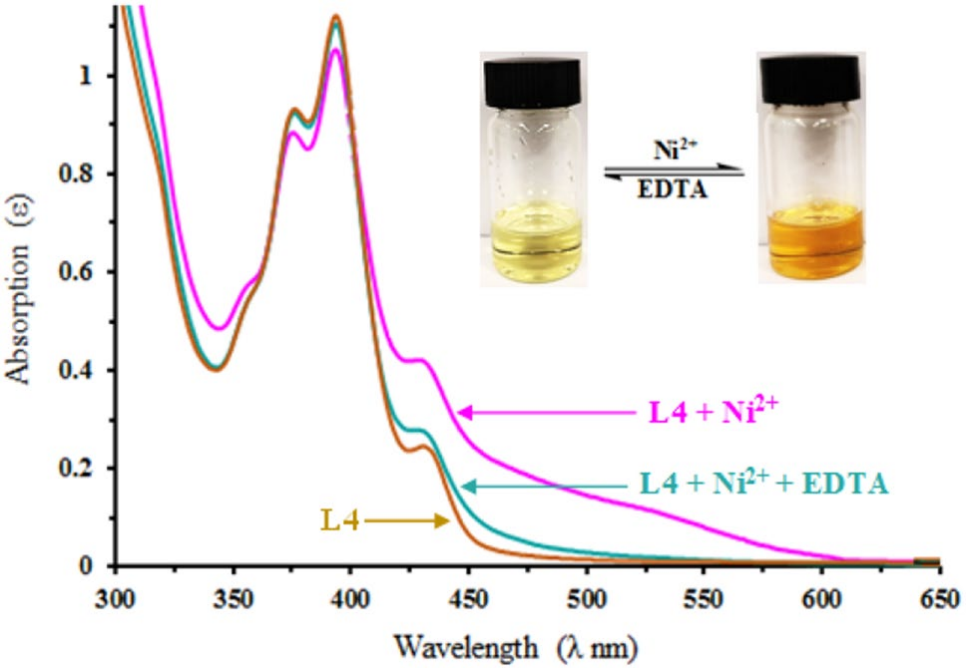
Changes in absorbance spectra of L4 (20 µM) in DMSO*–*H_2_O solution (1:2 v/v, HEPES buffer, pH = 7.4) in the presence of Ni^2+^ and EDTA.

The reversibility of receptor L4 towards Ni^2+^ was examined by adding ethylenediaminetetracetic acid (EDTA, 1 equiv.) to the complexed solution of L4 and Ni^2+^ (Fig. 7).

The solution’s color changed from red to light yellow (original color of L4). Upon addition of Ni^2+^ again the absorbance at 537 nm was recorded. The absorption changes in spectral bands were reversible even after several cycles with alternative sequential addition of Ni^2+^ and EDTA. These results indicate that L4 could be recyclable as an *off–on-off* receptor by the interaction of EDTA with L4–Ni^2+^ (Scheme 1). Such regeneration and reversibility could be valuable for the fabrication of Ni^2+^ sensors.

#### Synthesis of the L4–Ni^2+^ complex

**Scheme 2:**
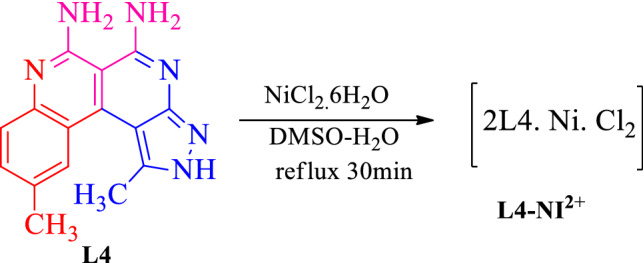
Preparative route for L4-Ni^2+^ complex.

The Ni^2+^ complex of receptor L4 was synthesized by mixing an Ni^2+^ salt with L4 using 1:2 ratio in DMSO-H_2_O solvent mixture. The yellow solution of the ligand immediately turned to red colored solution. The solution was further refluxed to get the solid product (Scheme 2).

Red solid, Yield: 84%, mp > 300 °C; λ_max_: 535 nm; λ_em_: 473 (λ_ex_ = 340); IR (ATR, cm^−1^): 3368, 3222, (NH), 1642, 1541, 1438, 1339, 1238, 1110, 1031, 1002, 825, 731; MS (ESI) m/z: 685.41 [2L4 + Ni + Cl_2_]^+^; Molar conductance: 0.41 S cm^2^ M^−1^; µ_eff_ (B.M.): 2.98.

**Figure 8:**
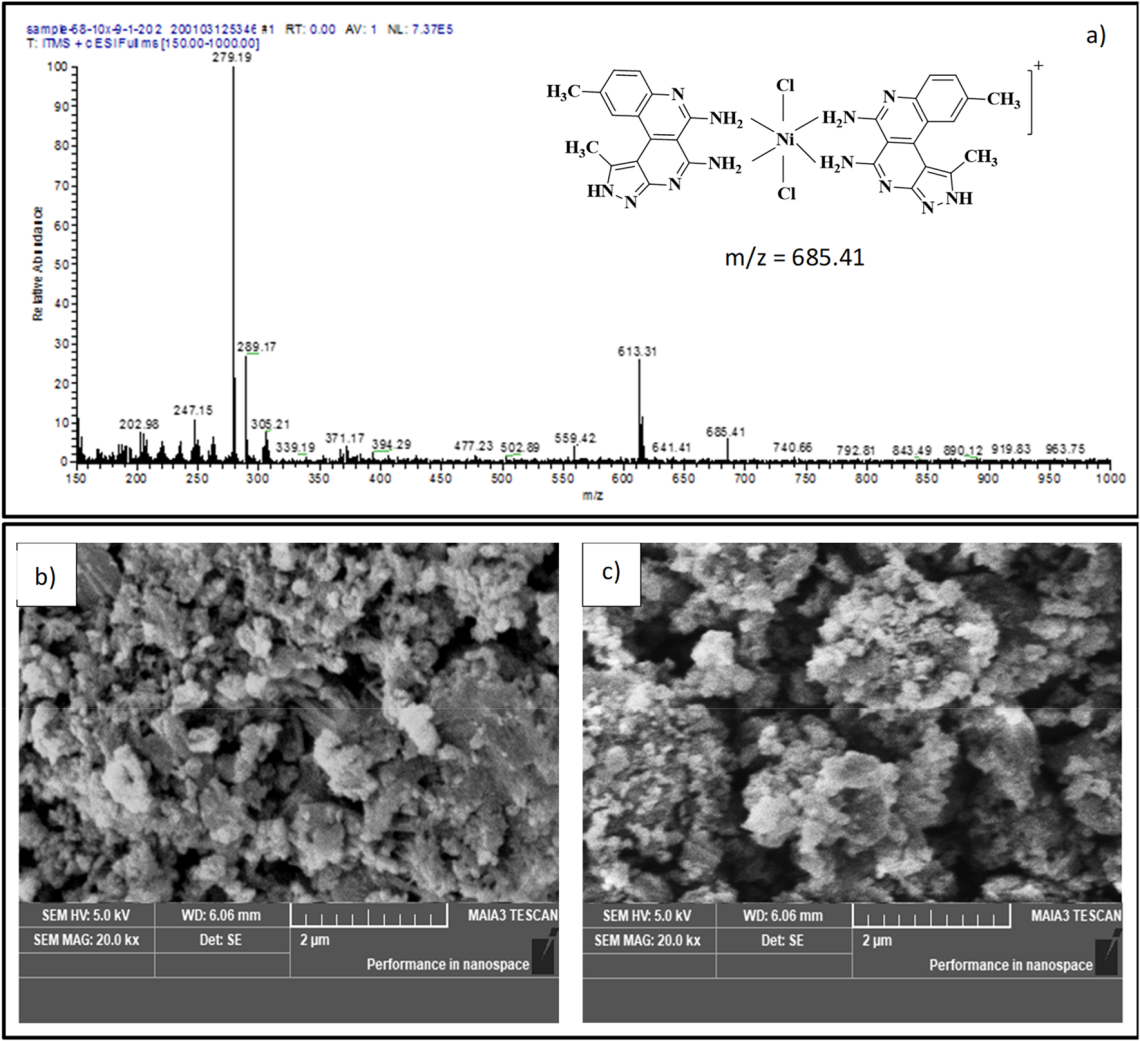
(**a**) ESIMS of L4-Ni^2+^ complex (**b**) The SEM images of Receptor L4 (**c**) L4-Ni^2+^ complex.

The synthesized complex of L4–Ni^2+^ was characterized using UV–Visible, fluorescence spectroscopy, molar conductance, SEM, ESI–MS and magnetic moment analysis. A 10^−3^ M solution of the L4–Ni^2+^ complex exhibited a molar conductance value of 0.4 S cm^2^ M^−1^ which suggests non-electrolyte behavior in DMSO solution. The ESI–MS of the synthesized L4–Ni^2+^ complex (Fig. 8a) showed the molecular ion peak at m/z 685.41 which matched very well with the calculated molar mass of [2L4 + Ni + Cl_2_ + H]. Furthermore, SEM analysis was carried out to obtain a better understanding of morphological difference before and after the addition of Ni^2+^ to L4 receptor (Fig. 8b-c). SEM images of receptor L4 show dense sprinkled elliptical shapes which are transformed into a rough stone like structure after complexation with Ni^2+^. Magnetic studies were used to confirm the geometry of the synthesized metal complex L4–Ni^2+^. The room temperature magnetic moment value of the solid complex is µ_eff_ (B.M.): 2.98 in line with an octahedral environment (2.9–3.3 B.M.) around the metal ion^47^. The proposed octahedral geometry is also supported by mass spectral analysis.

#### Practical application

In order to investigate the potential use of receptor L4 in real water samples, a calibration curve was drawn, which showed a good linear relationship (R2 = 0.9996, n = 3) between the absorbance of the L4–Ni^2+^ complex and the Ni^2+^ concentration (0–5 µM) at 537 nm (Fig. S19). The receptor L4 was used for the estimation of Ni^2+^ in drinking water, tap water and industrial waste water samples (Table 3). All water samples were analyzed in triplicate with good recoveries and RSD values. The results indicate that receptor L4 is highly specific and sensitive for Ni^2+^ estimations in environmental samples.

**Table 3:** Determination of Ni^2+^ in water samples.

Sample	Ni^2+^ added (µM)	Ni^2+^ found (µM)	Recovery (%)	RSD (n = 3) %
Drinking water	0.00	0.00		
	0.50^a^	0.47^b^ (0.50)^c^	94.0	2.13
	1.00^a^	0.98^b^ (1.00)^c^	98.0	1.97
	2.00^a^	1.97^b^ (2.00)^c^	98.5	1.21
Tap water	0.00	0.00		
	0.50^a^	0.46^b^ (0.50)^c^	92.0	2.43
	1.00^a^	0.97^b^ (1.00)^c^	97.0	2.04
	2.00^a^	1.96^b^ (2.00)^c^	98.0	1.68
Industrial waste water	0.00	0.00		
	0.50^a^	0.45^b^ (0.50)^c^	90.0	2.89
	1.00^a^	0.96^b^ (0.99)^c^	96.0	3.04
	2.00^a^	1.95^b^ (1.99)^c^	97.5	1.89

^a^Artificially spiked concentration of Ni^2+^.

^b^Results obtained by newly synthesized receptor L4.

(·)^c^Results obtained from ICP-OES analysis.

**Figure 9:**
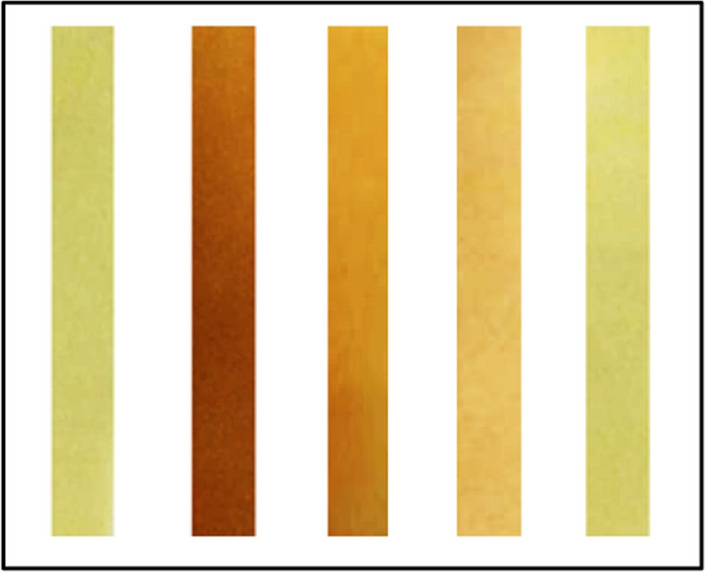
Color change of the test strips of L4 (1 × 10^−3^ M) at various concentrations of Ni^2+^ in water, from left to right: 0, 1 × 10^−3^ M, 1 × 10^−4^ M, 1 × 10^−5^ M and 1 × 10^−6^ M.

To explore another application of receptor L4, test kits were prepared by immersing filter paper in receptor L4 (1 × 10^−3^ M, HEPES buffer, pH = 7.4) and then air drying to investigate a “dip-stick” method for detection of Ni^2+^. When the prepared test strips were immersed into aqueous solutions of Ni^2+^ with different concentrations, clear color changes from yellow to red were observed (Fig. 9). The results showed that discernible concentrations of Ni^2+^ can be as low as 1 × 10^−5^ M. The “dip-stick” method did not require any additional equipment for detection of Ni^2+^ and should be highy attractive for “in-the-field” measurements.

### Theoretical details on structural aspects of L4–Ni^2+^

#### Computational details

In order to evaluate the interaction between L4 and Ni^2+^ theoretical calculations on the L4-NiCl_2_ complex were performed. Geometry optimization calculations were performed for the free species L4 and NiCl_2_, and complexes L4-NiCl_2_ and 2L4-Ni_2_Cl_2_ (with two L4 molecules). The calculations employed the DFT method by applying the M06L^48^ functional and def2-SVP^49^ basis set. The calculations were carried out with the Gaussian 09^50^ package (Revision D.01). To evaluate the interactions in the complexes we performed Atoms in Molecules^51–53^ (AIM) analysis. The AIM analyses were carried out with Multiwfn^54^ package from single-point calculations with M06L functional and def2-TZVP basis set with SMD solvent model with DMSO as solvent and visualized with VMD^55^. Frontier Molecular Orbitals (FMO) analyses were performed to investigate the effect of L4 complexation with Ni^2+^. The FMO analyses were carried out with TD-DFT single-point calculations (from their respective optimized geometries) by applying the M06-2X^56^/def2-TZVP^49^ level of theory with the SMD solvent model and DMSO as solvent in the Orca^57^ package.

#### Theoretical calculations

**Figure 10:**
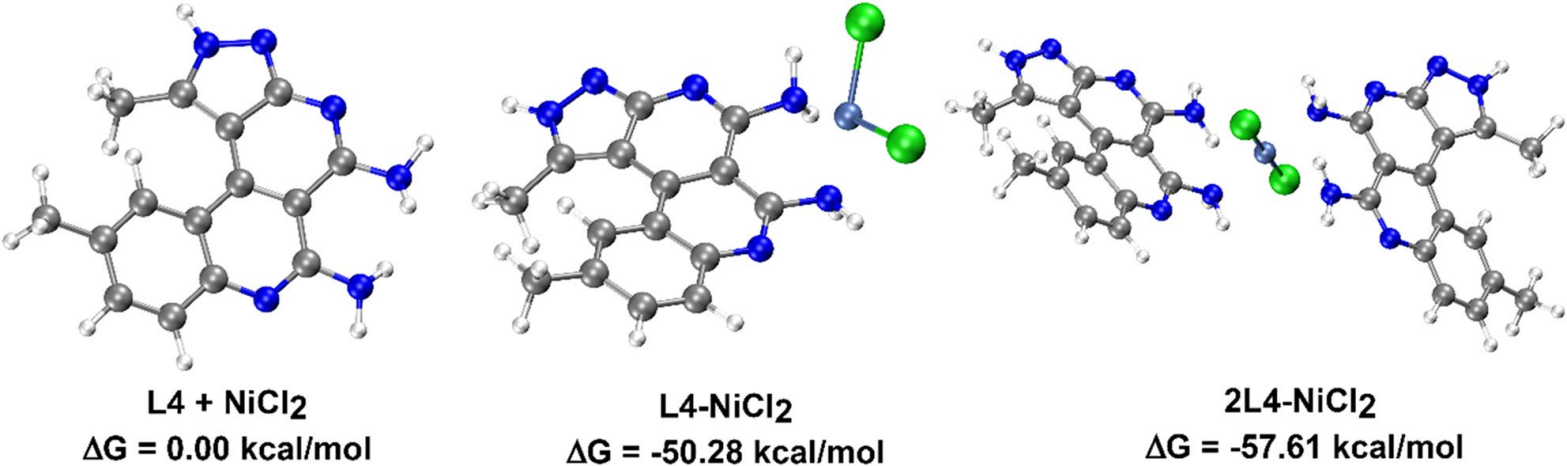
Scheme of optimized geometries (with M06L/def2-SVP method) for L4-NiCl_2_ complexes and 2L4-NiCl_2_ complexes with Ni^2+^ with their respective relative Gibb free energies.

The experimental results indicated complex formation with 2:1 stoichiometry between L4 and NiCl_2_. Thus, we performed calculations for free L4 and NiCl_2_, for L4-NiCl_2_ complexes (with one L4 molecule) and for 2L4-NiCl_2_ complexes (with two L4 molecules) as shown in Fig. 10. The relative energies are shown in Table S2 and demonstrate that the complexation between L4 and NiCl_2_ is spontaneous. The energies for complexation with one or two L4 molecules showed that the complex with two L4 molecules is more stable than with just one molecule in agreement with experimental results. Thus, the FMO and AIM analyses were performed just for complexes with two L4 molecules.

#### FMO and GRD analysis

**Figure 11:**
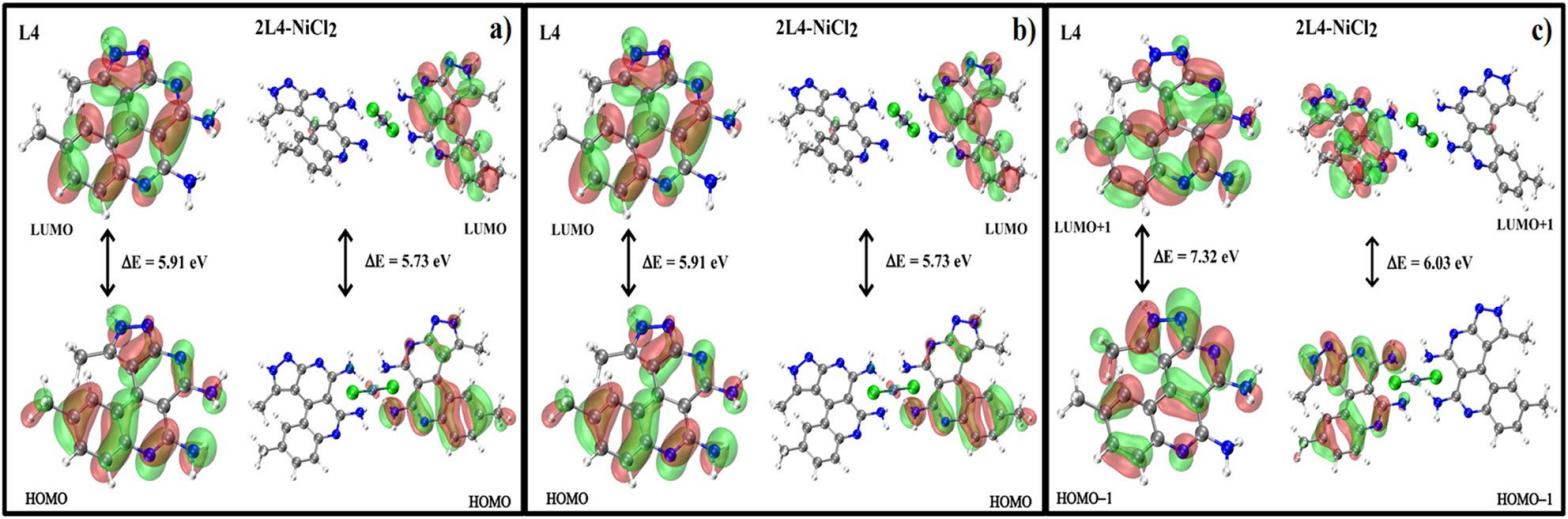
(**a**) FMO surfaces plot. HOMO and LUMO for L4 (left) and 2L4-NiCl_2_ (right), (**b**) FMO surfaces plot. HOMO-1 and LUMO + 1 for L4 (left) and 2L4-NiCl_2_ (right), (**c**) FMO surfaces plot. HOMO-2 and LUMO + 2 for L4 (left) and 2L4-NiCl_2_ (right).

The energies for frontier molecular orbitals of free L4 and the 2L4-NiCl_2_ complex are shown in Table S3. It was verified through the FMO analysis species that the complexation between L4 and NiCl_2_ causes a slight decrease of the frontier molecular orbitals and the 2L4-NiCl_2_ complex showed lower HOMO/LUMO energy gaps, particularly for HOMO-1/LUMO + 1 and HOMO-2/LUMO + 2 which are related to the interaction with nickel. The surfaces for frontier molecular orbitals of L4 and 2L4-NiCl_2_ are shown in Fig. 11.

#### AIM analysis

**Figure 12:**
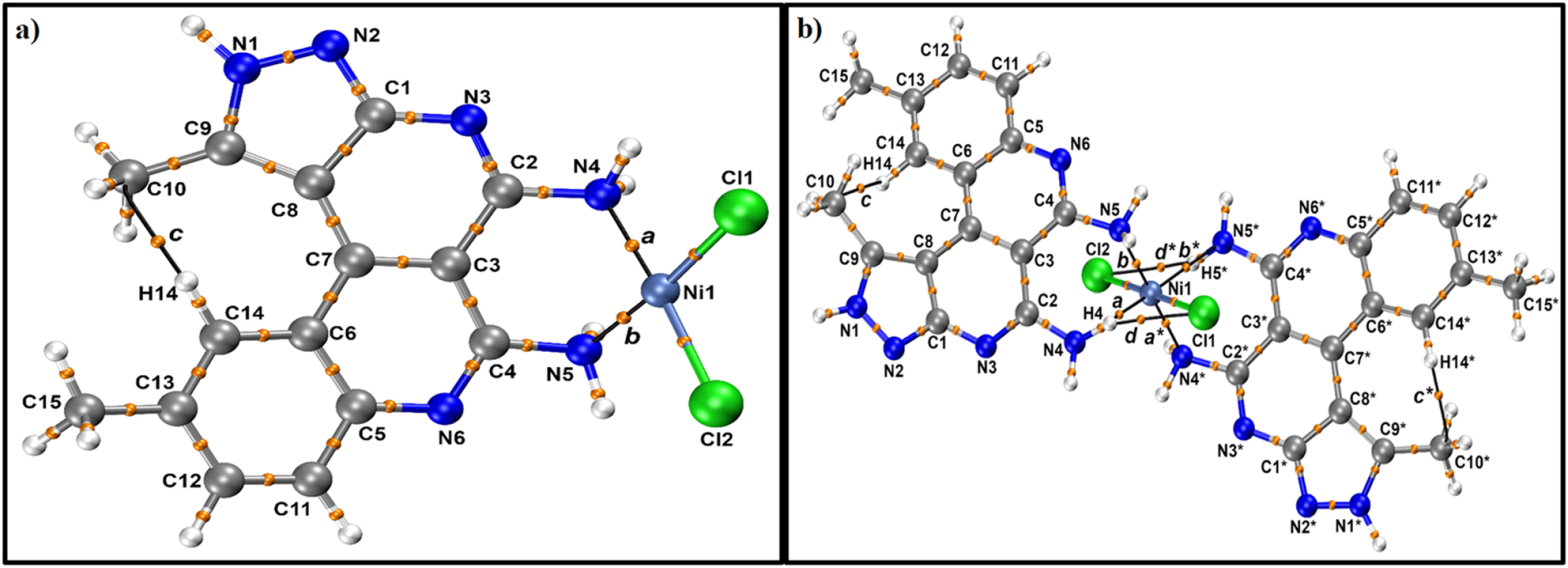
(**a**) AIM molecular graph for L4-NiCl_2_. Bond Critical Points (orange spheres) and Bond Paths (solid dashed black lines), (**b**) AIM molecular graph for 2L4-NiCl_2_ Bond Critical Points (orange spheres) and Bond Paths (solid dashed black lines).

The AIM properties for the 2L4-NiCl_2_ complexes are shown in the Table S4 and their respective molecular graphs are shown in Fig. 12a-b respectively. The characterization of strengthening of the interaction between L4 and NiCl_2_ was performed through the topological analysis of the electronic density. Therefore, the presence of an interaction between atoms was featured by the presence of a Bond Path (BP) between two attractor (atoms in a molecule) and their characteristics such covalence and strength were determined though the AIM properties in their Bond Critical Points (BCPs). The main AIM properties that were evaluated in this work were the electronic density, *ρ*(r), Laplacian of density, ∇^2^*ρ*(r), and density of potential energy, *V*(r).

Thus, the presence of the interaction between NH_2_ and Ni was revealed by the presence of a BP between N and Ni atoms. The AIM molecular graphs with their respective labels are shown in Fig. 12a-b. The BCPs ***a*** and ***b*** are related to the Ni–N bonds in the complexes L4-NiCl_2_ and 2L4-NiCl_2_. The complexes’ formation showed BCPs *a* and *b* with positive value of ∇^2^*ρ*(r) with can indicate a non-covalent interaction or ionic bond. When the values of electronic density, *ρ*(r), were analyzed for these BCPs was verified that the BCPs *a* for both complexes showed high negative values of density of potential energy, *V*(r), which indicates that the bonds related to BCPs *a* have the characteristic of ionic bonds. The BCP b L4-NiCl_2_ also showed an ionic characteristic. However, the 2L4-NiCl_2_ complex showed the weakening of one of their Ni-NH_2_ interaction to formation of another Ni-NH_2_ interaction with the second L4 molecule. Thus, the 2L4-NiCl_2_ showed two ionic bonds between N-Ni (BCPs *a* and b*) with ρ(r) = 0.0922 a.u. and *ρ*(r) = 0.0901 a.u., respectively, and two non-covalent interactions between N-Ni (BCPs *b* and a*), that were revealed by the presence of ∇^2^*ρ*(r) > 0, though, with low values of *ρ*(r) (*ρ*(r) = 0.0202 a.u. and *ρ*(r) = 0.0181 a.u., respectively) and low negative values of *V*(r). While the L4-NiCl_2_ complex showed two ionic bonds with the same L4 with BCPs a and *b*, the 2L4-NiCl_2_ complex showed two ionic bonds and several non-covalent interactions (BCPs *c*, *d*, c* and d*) between L4 and NiCl_2_, Thus, such non-covalent interactions should be related with the stabilization of 2L4-NiCl_2_ complex in relation to L4-NiCl_2_. Beyond the interaction between N atoms from L4 and Ni^2+^ was verified the presence of NH···Cl interaction (BCPs *d* and d* with *ρ*(r) = 0.0165 a.u. and *ρ*(r) = 0.0133, respectively) in the 2L4-NiCl_2_ complex which should increase the NH stretching frequencies shift in relation to free L4 which agree with experimental results.

## Conclusion

In summary, we have successfully characterized the photophysical properties of benzo[*c*]pyrazolo[2,7]naphthyridines (L1-L4) which were prepared by a green synthetic route. The receptors (L1-L4) allow selective and sensitive sensing of Ni^2+^ in aqueous media over a wide range of pH (4–11) even in the presence of competitive ions i.e. Fe^3+^, Cu^2+^, Co^2+^. A unique colorimetric response to Ni^2+^ is observed (yellow to red) through coordination of receptor and Ni^2+^ complex could be recyclable through treatment with EDTA. The detection limit of Ni^2+^ was found to be in range of 0.2–0.5 µM for (L1-L4) by UV–Visible data, and 0.040–0.47 µM by fluorescence data, which is lower than the permissible value of Ni^2+^ (1.2 µM) in drinking water and opens a potential application of the receptors in recognizing Ni^2+^ in environment. The binding stoichiometries of (L1-L4) with Ni^2+^ were found to be 2:1 through Job’s plot and ESI–MS analysis. The fluorescence properties of the receptors were evaluated with fluorescence quenching by coordination with Ni^2+^. As a practical application, the most efficient receptor L4 could be used to quantify and detect Ni^2+^ in real water samples and also applied for fabrication of test kits using the “dip-stick” method. To the best of our knowledge, receptors (L1-L4) are the first reported multifunctional, naked eye chemosensors for sensing of Ni^2+^ in aqueous solutions. Theoretical calculations demonstrate that the stoichiometry of complexation between L4 and NiCl_2_ should be 2:1 as experimentally verified. The non-covalent interactions between L4 and NiCl_2_ in the formed complex, particularly involving the –NH_2_ groups can broaden the NH stretching frequencies, which is consistent with the experimental results. The FMO demonstrate the complexation with Ni decreases the frontier molecular orbitals and their respective energy gaps.

## Experimental

### Materials and equipment

All the solvents and reagents used for synthesis were of analytical grade and used as received. Infrared (IR) spectra were recorded by Bruker Alpha FT-IR spectrophotometer. Mass spectra were recorded by a Thermo Scientific LTQ-XL system fitted with electrospray ionization (ESI) source, Jeol 600 MS Route, and Jeol Hx110 mass spectrometer (EI-HR). Pre-coated aluminum sheets of silica gel 60 GF254 (Merck) were used as TLC plates to check the purity of compounds. Quantitative determination of nickel was carried out by Inductively Coupled Plasma-Optical Emission Spectrometer (iCAP6500 ICP-OES, Thermo Scientific, Cambridge, United Kingdom). The pH was measured using a Metrohm, 78 l pH/ion meter. Magnetic moments were estimated using a magnetic susceptibility balance (Sherwood Auto, 2005) at room temperature. Metal conductance of metal complexes was recorded by using an InoLab IDS Multi9430 conductivity meter.

### Synthesis of receptors (L1–L4)

The synthesis of receptors (L1–L4) was carried out in two steps following our previously reported protocol^37^.

### Synthesis of L4-Ni^2+^ complex

The DMSO (3 ml) solution of L4 (0.2 mmol) and 2 ml water solution of NiCl_2_.6H_2_O (0.12 mmol) were mixed and stirred at room temperature for 30 min. The yellow solution of receptor L4 immediately turned to red colored solid product, which was filtered and washed with distilled water.

### UV–visible and fluorescence titrations

Stock solutions of receptors, L1–L4 (1 × 10^−3^ M) were prepared in DMSO–H_2_O (v/v = 1:2) using HEPES buffer solution (pH = 7.4). Stock solutions of different guest metal ions (1 × 10^−3^ M) were prepared by using chloride salts of the respective metals (Al^3+^, Ca^2+^, Cd^2+^, Co^2+^, Cr^3+^, Cu^2+^, Fe^3+^, Hg^2+^, K^+^, Mg^2+^, Mn^2+^, Na^+^, Ni^2+^, Pb^2+^, Sr^2+^ and Sn^2+^) and the stock solutions (1 × 10^−4^ M) of different anions like Cl^−^, I^−^, Br^−^, CN^−^, ClO_4_^−^, F^−^, HSO_4_^−^, AcO^−^ and SCN^−^ from TBA salts were prepared in deionized water. For ratiometric titrations, the solutions of various concentrations of receptor with increasing concentration of cations were prepared separately.

### Competition experiments

For Ni^2+^ the stock solution of receptor L4 (1 × 10^−3^ M) was prepared in DMSO–H_2_O (v/v 1:2) using HEPES buffer of pH = 7.4. Stock solution of different guest cations (1 × 10^−3^ M) were prepared in water and added to 4 mL of the solution of receptor L4 to give 10 equivalents of metal ions. Then Ni^2+^ solution was added into mixed solutions of each metal ion to make 1 equivalent. A few minutes after mixing, the UV–visible spectra were recorded at room temperature.

### Water sample collection and Ni^2+^ determination

The drinking water samples, tap water and industrial waste water samples were collected, preserved and stored in plastic containers for Ni^2+^ analysis. Industrial waste water samples were filtered prior to analysis. Each sample was analyzed in triplicate using receptor L4 and ICP-OES as standard method (Table 3). Spiking and recovery method was used in order to validate chemosensing performance of our newly developed sensor L4. UV–visible spectral measurement of water samples containing Ni^2+^ was carried out by adding 0.5 mL of receptor L4 to 2.5 mL of sample solutions and pH of solution was maintained at 7.4 using HEPES buffer. The solutions were allowed to stand for 10 min at room temperature and absorption measurements were taken at 537 nm. Filtered water samples were directly used for ICP-OES analysis.

### Colorimetric test strips

The test kits were prepared by immersing filter paper strips in to receptor L4 solution 1 × 10^−3^ M (DMSO–H_2_O (v/v 1:2) using HEPES buffer of pH = 7.4) and then dried in air. Then the pure water solution with different Ni^2+^ concentrations were prepared and the prepared test strips were immersed in water samples and color change from yellow to red was observed.

### Magnetic moments

Magnetic moments of the prepared solid complex were estimated by magnetic susceptibility balance Sherwood (Auto, 2005) at room temperature.

## Supplementary Information

Supplementary Information.
