# Supplementary material for: Naphthyridine derived colorimetric and fluorescent turn off sensors for Ni2+ in aqueous media
Source: Sci Rep. 2021 Sep 28;11:19242. doi: 10.1038/s41598-021-98400-2 (PMC8479070; doi:10.1038/s41598-021-98400-2)
Supplement: Supplementary file 1 — Supplementary Information. [file 41598_2021_98400_MOESM1_ESM.docx]

**Supporting Information**

**Naphthyridine derived colorimetric and fluorescent *turn off* sensors for Ni^2+^ in aqueous media**

Abida Ashraf,^a^ Muhammad Islam,^a^ Muhammad Khalid*,^b^ Anthony P. Davis,^c^ Muhammad Tayyeb Ahsan,^a^ Muhammad Yaqub,^a^ Asad Syed,^d^ Abdallah M. Elgorban ^d^ Ali H, Bahkali,^d^ and Zahid Shafiq*,^a^

*^a^ Institute of Chemical Sciences, Bahauddin Zakariya University, Multan Pakistan*

*^b^Department of Chemistry, Khwaja Fareed University of Engineering and Information Technology, Rahim Yar Khan, 64200, Pakistan*

*^c^ School of Chemistry, University of Bristol, Cantock’s Close, Bristol BS8 1TS, UK*

*^d^ Department of Botany and Microbiology, College of Science, King Saud University, P.O. 2455, Riyadh, 11451, Saudi Arabia*

**General procedure for one-pot multi-component synthesis of naphthyridines (L1-L4)**

The synthesis of receptors **(L1-L4)** were carried out by following our previously reported protocol[1] which is outlined below,

A mixture of isatin **6** (2 mmol), malononitrile **7** (2 mmol), 3-amino-5-methylpyrazole **8** (2 mmol) were fused, refluxed in 5 ml H_2_O for appropriate time (4-5 h) to form intermediate spiroindoline scaffolds. After consumption of the reactant, as indicated by TLC, NaOH (0.6 equiv.) was added to the crude product. The reaction mixture was further refluxed for 2-3 hours. The reaction was allowed to cool down to room temperature. The precipitate formed was filtered by suction and washed thoroughly with water/ethanol (1:1) to obtain the crude product. The resulting residue was recrystallized in methanol to obtain pure heterocyclic receptor **L1-L4**.

**Scheme S1:** One-pot multicomponent synthesis of benzo[*c*]pyrazolo[2,7]naphthyridines **L1-L4.**

**1-methyl-2H-benzo[c]pyrazolo[4,3-f][2,7]naphthyridine-5,6-diamine (L1)**

Yellow solid, Yield: 81%, mp > 300 ^օ^C^1^; IR (ATR, cm^-1^): 3420, 3295, 3104 (NH), 1613, 1541, 1385, 1310,839, 746; MS (ESI) m/z: 265.17 [M+H]^+^. Anal. Calcd for C_14_H_12_N_6_: C, 63.62; H, 4.58; N, 31.80%; found: C, 63.59; H, 4.56; N, 31.76%

**10-fluoro-1-methyl-2H-benzo[c]pyrazolo[4,3-f][2,7]naphthyridine-5,6-diamine (L2)**

Yellow solid, Yield: 84% , mp > 300 ^օ^C^1^; IR (ATR, cm^-1^): 3414, 3335, 3086 (NH), 1619, 1602, 1548, 1496, 1386, 1244, 1172, 976, 849, 820, 753; MS (ESI) m/z: 283.17 [M+H]^+^. Anal. Calcd for C_14_H_11_FN_6_: C, 59.57; H, 3.93; N, 29.77%; found: C, 59.52; H, 3.89; N, 29.72%.

**1-methyl-10-(trifluoromethoxy)-2H-benzo[c]pyrazolo[4,3-f][2,7]naphthyridine-5,6-diamine (L3)**

Yellow solid, Yield: 85%, mp > 300 ^օ^C^1^; IR (ATR, cm^-1^): 3405, 3300, 3109 (NH), 1614, 1548, 1386, 1204, 1147, 827, 696; MS (ESI) m/z: 349.17 [M+H]^+^. Anal. Calcd for C_15_H_11_F_3_N_6_O: C, 51.73; H, 3.18; N, 24.13%; found: C, 51.68; H, 3.16; N, 24.10%.

**1,10-dimethyl-2H-benzo[c]pyrazolo[4,3-f][2,7]naphthyridine-5,6-diamine (L4)**

Yellow solid, Yield: 83%, mp > 300 ºC^1^; IR (ATR, cm^-1^): 3421, 3290, 3206, 3108 (NH), 1612, 1570, 1504, 1380, 1317, 1100, 1002, 840, 746, 701, 664, 603; MS (ESI) m/z: 279.17 [M+H]^+^. Anal. Calcd for C_15_H_14_N_6_: C, 64.73; H, 5.07; N, 30.20%; found: C, 64.68; H, 5.04; N, 30.14%.


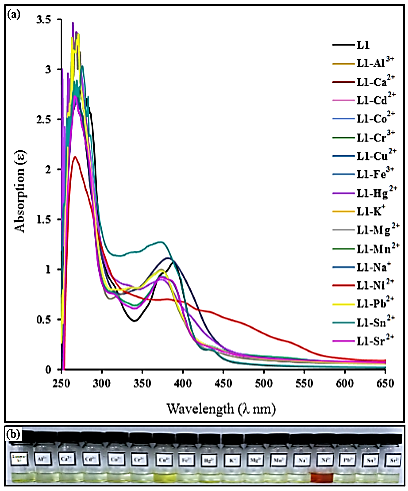


**Figure S1**: **(a)** Absorption spectral changes of **L1**  (20 µM) in the presence of different metal ions in DMSO–H2O (v/v 1:2, HEPES buffer pH = 7.4). **(b)** Visual colorimetric responses of receptors **L1** upon addition of one equivalent various metal ions.


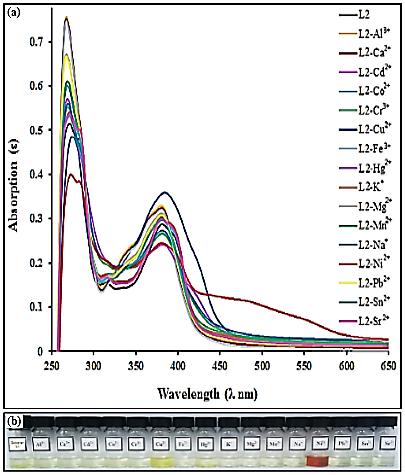


**Figure S2**: **(a)** Absorption spectral changes of **L2** (20 µM) in the presence of different metal ions in DMSO–H2O (v/v 1:2, HEPES buffer pH = 7.4). **(b)** Visual colorimetric responses of receptors **L2** upon addition of one equivalent various metal ions.


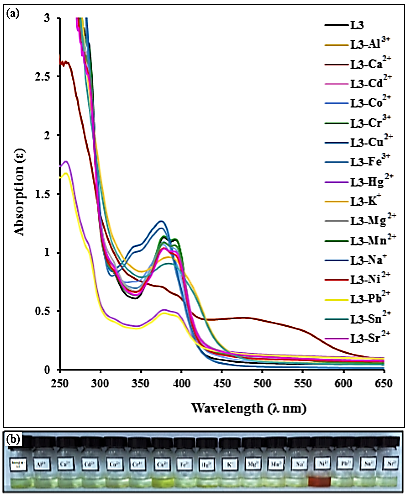


**Figure S3** **(a)** Absorption spectral changes of **L3** (20 µM) in the presence of different metal ions in DMSO–H2O (v/v 1:2, HEPES buffer pH = 7.4). **(b)** Visual colorimetric responses of receptors **L3** upon addition of one equivalent various metal ions.


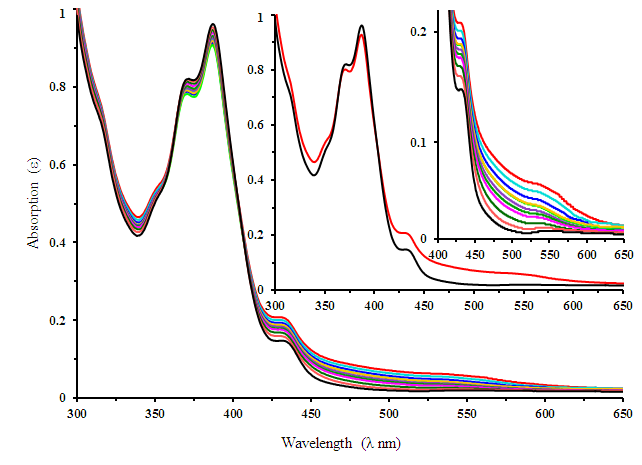


**Figure S4**: Absorbance titration spectra of receptor **L1** (20 µM) in the presence of various concentrations of Ni^2+^ in DMSO:H_2_O (v/v 1:2, HEPES buffer, pH = 7.4).


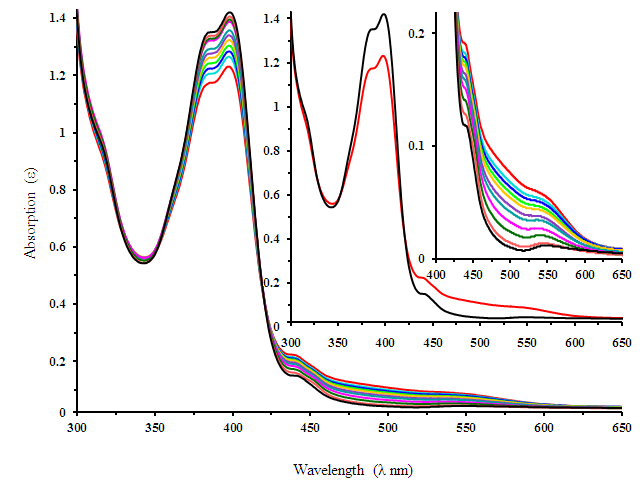


**Figure S5**: Absorbance titration spectra of receptor **L2** (20 µM) in the presence of various concentrations of Ni^2+^ in DMSO:H_2_O (v/v 1:2, HEPES buffer, pH = 7.4).


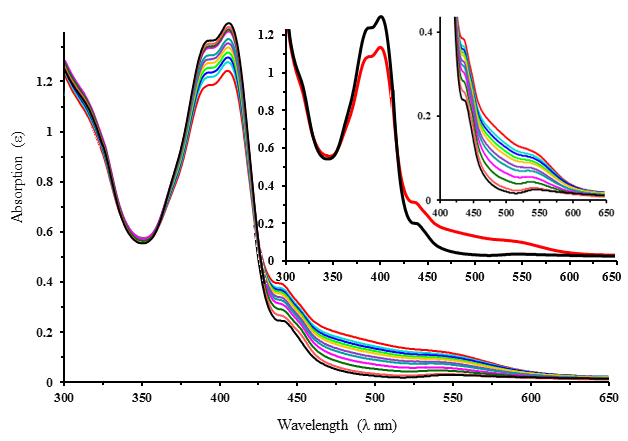


**Figure S6**: Absorbance titration spectra of receptor **L3** (20 µM) in the presence of various concentrations of Ni^2+^ in DMSO:H_2_O (v/v 1:2, HEPES buffer, pH = 7.4).


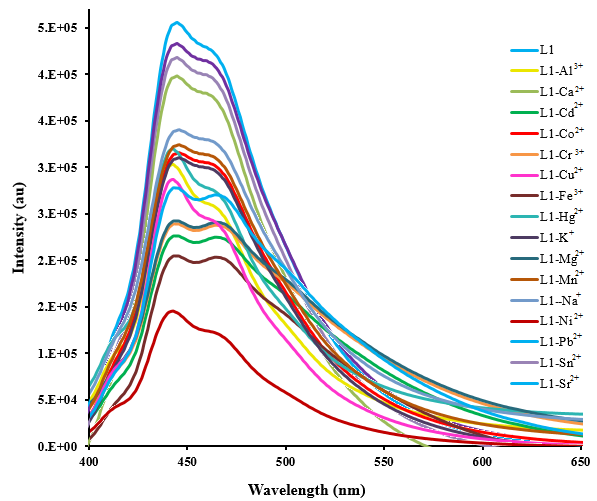


**Figure S7** : Fluorescence spectral changes of **L1** (20 µM) in the presence of different metal ions in DMSO–H_2_O (v/v 1:2, HEPES buffer pH = 7.4), (λ_ex_ = 340 nm).


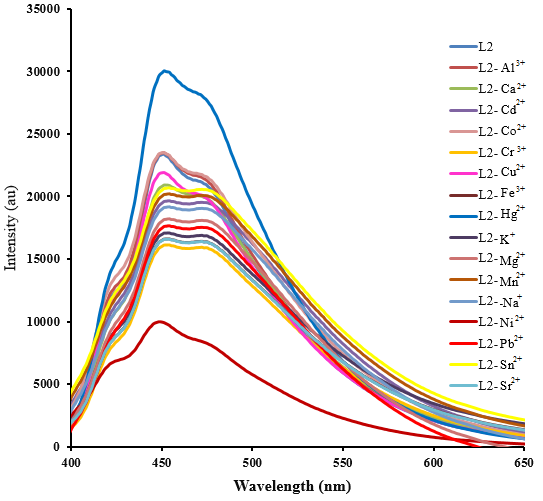


**Figure S8** : Fluorescence spectral changes of **L2** (20 µM) in the presence of different metal ions in DMSO–H_2_O (v/v 1:2, HEPES buffer pH = 7.4), (λ_ex_ = 380 nm).


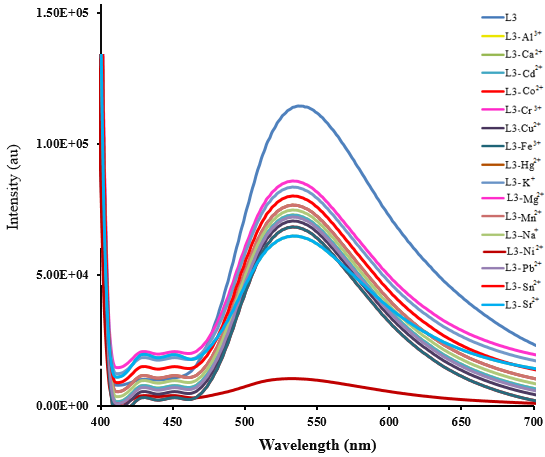


**Figure S9** : Fluorescence spectral changes of **L3** (20 µM) in the presence of different metal ions in DMSO–H_2_O (v/v 1:2, HEPES buffer pH = 7.4), (λ_ex_ = 490 nm).


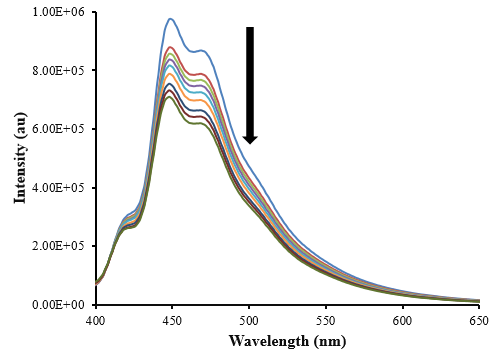


**Figure S10**: Fluorescence titration spectra of receptor **L1** (20 µM) in the presence of various concentrations of Ni^2+^ in DMSO:H_2_O (v/v 1:2, HEPES buffer, pH = 7.4). (λ_ex_ = 340 nm).


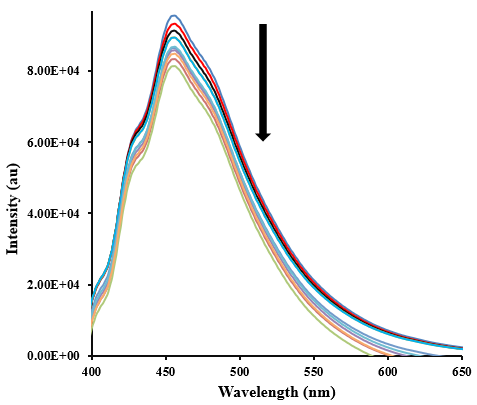


**Figure S11**: Fluorescence titration spectra of receptor **L2** (20 µM) in the presence of various concentrations of Ni^2+^ in DMSO:H_2_O (v/v 1:2, HEPES buffer, pH = 7.4). (λ_ex_ = 380 nm).


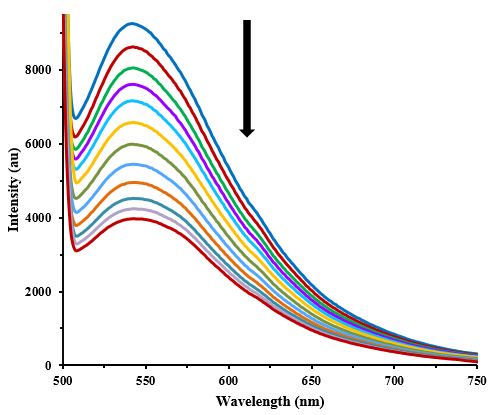


**Figure S12**: Fluorescence titration spectra of receptor **L3** (20 µM) in the presence of various concentrations of Ni^2+^ in DMSO:H_2_O (v/v 1:2, HEPES buffer, pH = 7.4). (λ_ex_ = 490 nm).


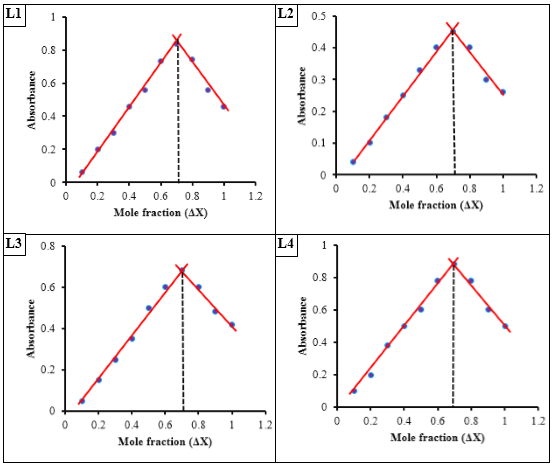


**Figure S13**: Job's plot of **L1-L4** and Ni^2+^ in DMSO–H_2_O solution (v/v 1:2, HEPES buffer, pH = 7.4)


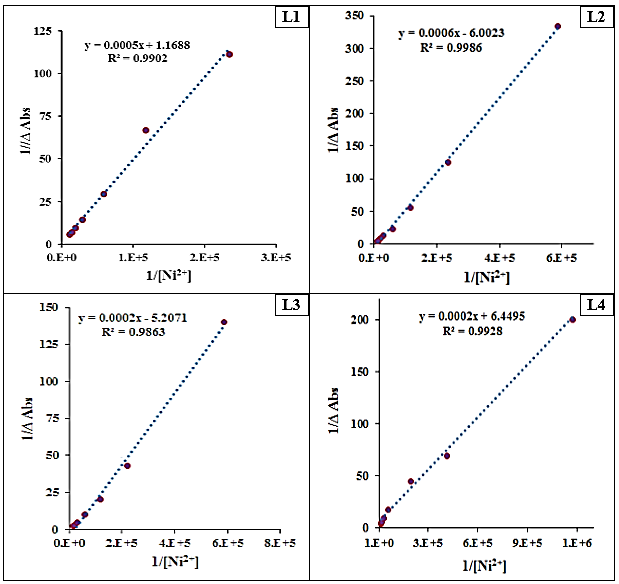


**Figure S14**: Benesi-Hildebrand plots of UV-visible titration results for L1-L4 with Ni^2+^ ions in DMSO-H_2_O (1:2 v/v, HEPES buffer pH = 7.4).

**Figure S15**: Benesi-Hildebrand plots of fluorescence titration results for L1-L4 with Ni^2+^ ions in DMSO-H_2_O (1:2 v/v, HEPES buffer pH = 7.4).


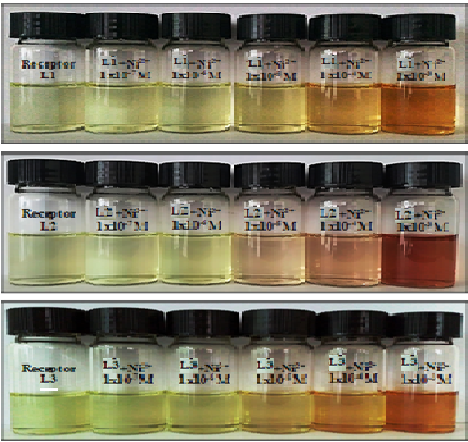


**Figure S16**: Naked eye detection limit for receptor **L1-L3**.


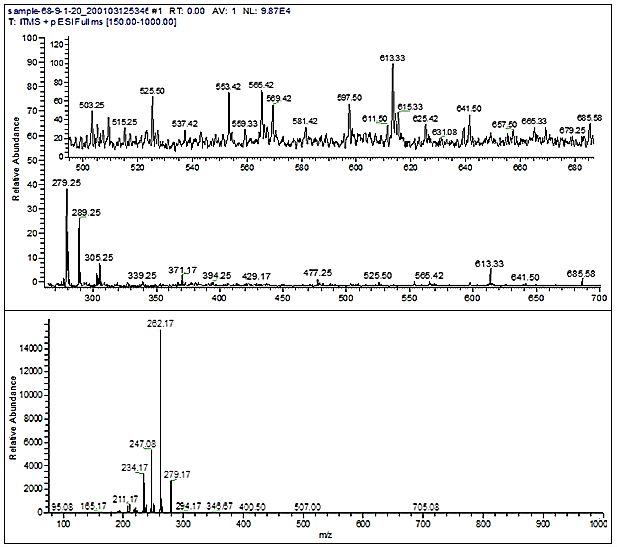


**Figure S17**: Positive-ion electrospray ionization mass spectrum of L4 (20 µM) upon addition of Ni^2+^ (10 µM)


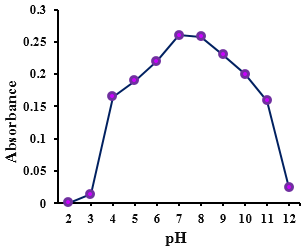


**Figure S18**: Absorbance of **L4** at 537 nm in DMSO–H_2_O (v/v 1:2) with different pH

**(b)**

**Figure S19**: L1 absorbance at 535 nm for L1 as a function of Ni^2+^ ion concentration,

L2 absorbance at 538 nm for L2 as a function of Ni^2+^ ion concentration,

L3 absorbance at 550 nm for L3 as a function of Ni^2+^ ion concentration,

L4 absorbance at 537 nm for L4 as a function of Ni^2+^ ion concentration.

**Table S1**: Association constant values of receptor **L1-L4** with nickel.

| **Receptor** | **Association constant**  **M^-2^** | |
| --- | --- | --- |
|  | **Absorbance** | **Emission** |
| **L1** | 2.3 x 10^3^ | 7.3 x 10^3^ |
| **L2** | 1.0 x 10^4^ | 1.1 x 10^5^ |
| **L3** | 2.6 x 10^4^ | 1.9 x 10^5^ |
| **L4** | 3.2 x 10^4^ | 3.2 x 10^5^ |

**Table S2:** Thermochemical data for **L4** and their respective complexes with NiCl_2_. Relative electronic energies (with zero-point correction), enthalpies and Gibbs free energies. All energies relative to free **L4** and NiCl_2_.

| **Structure** | **ΔE+ZPE (kcal/mol)** | **ΔH (kcal/mol)** | **ΔG (kcal/mol)** |
| --- | --- | --- | --- |
| L4 + NiCl_2_ | 0.00 | 0.00 | 0.00 |
| L4-NiCl_2_ | -61.72 | -62.21 | -50.28 |
| 2L4-NiCl_2_ | -81.98 | -81.87 | -57.61 |

**Table S3:** Energies for the frontier molecular orbitals (LUMO, HOMO, LUMO+1, HOMO-1, LUMO+2, HOMO-2) and their respective gap energies (Δ*E*) for free **L4** and **2L4-NiCl_2_** complex (two **L4** molecules). All MO energies are given in *eV.*

|  | **L4** | |  | **2L4-NiCl_2_** | |
| --- | --- | --- | --- | --- | --- |
| MOs | *Energy* | Δ*E* | MOs | *Energy* | Δ*E* |
| LUMO | -1.12 |  | LUMO | -1.59 |  |
| HOMO | -7.02 | 5.91 | HOMO | -7.32 | 5.73 |
| LUMO+1 | -0.09 |  | LUMO+1 | -1.52 |  |
| HOMO-1 | -7.41 | 7.32 | HOMO-1 | -7.55 | 6.03 |
| LUMO+2 | 0.75 |  | LUMO+2 | -0.90 |  |
| HOMO-2 | -7.95 | 8.70 | HOMO-2 | -7.77 | 6.87 |

**Table S4:** AIM properties for the interactions between Ni and **L4** and non-covalent interaction in these systems calculated with M06L/def2-TZVP with DMSO as solvent (SMD). Electronic density ($\rho$(r)), Laplacian of density ($\nabla^{2}\rho$(r)), ellipticity ($\varepsilon$) and density of potential energy ($V$(r)), all in atomic units, and Binding Energies (BE) in kcal/mol, calculated though the equation of Espinosa^58^, BE = V(r)/2.

| **Complex** | **Bond** | **BCP** | ***ρ*(r)** | **𝛻^2^*ρ*(r)** | ***V*(r)** | ***H*(r)** | **ε** | **BE** |
| --- | --- | --- | --- | --- | --- | --- | --- | --- |
|  | N4–Ni1 | ***a*** | 0.0931 | 0.4081 | -0.1489 | -0.0235 | 0.0843 | -46.73 |
| **L4-NiCl_2_** | N5–Ni1 | ***b*** | 0.0940 | 0.4078 | -0.1501 | -0.0241 | 0.0814 | -47.11 |
|  | C10–H14 | ***c*** | 0.0133 | 0.0499 | -0.0085 | 0.0020 | 0.8785 | -2.68 |
| **2L4-NiCl_2_** | N4–Ni1 | ***a*** | 0.0922 | 0.4064 | -0.1475 | -0.0229 | 0.1108 | -46.26 |
|  | N5–Ni1 | ***b*** | 0.0202 | 0.0625 | -0.0169 | -0.0007 | 0.4313 | -5.32 |
|  | C10–H14 | ***c*** | 0.0133 | 0.0503 | -0.0087 | 0.0019 | 0.8237 | -2.72 |
|  | Cl1–H4 | ***d*** | 0.0165 | 0.0583 | -0.0102 | 0.0022 | 0.6972 | -3.20 |
|  | N4*–Ni1 | ***a**** | 0.0181 | 0.0578 | -0.0147 | -0.0001 | 1.1648 | -4.60 |
|  | N5*–Ni1 | ***b**** | 0.0901 | 0.3950 | -0.1431 | -0.0222 | 0.1067 | -44.90 |
|  | C10*–H14* | ***c**** | 0.0178 | 0.0598 | -0.0106 | 0.0022 | 0.6670 | -3.32 |
|  | Cl2–H4* | ***d**** | 0.0133 | 0.0501 | -0.0086 | 0.0019 | 0.8192 | -2.71 |

**References:**

1. Ashraf, A., Shafiq, Z., Mahmood, K., Yaqub, M. & Rauf, W. Regioselective, one-pot, multi-component, green synthesis of substituted benzo [c] pyrazolo [2, 7] naphthyridines. *RSC Advances* **10**, 5938-5950 (2020).
